# NOCICEPTOR NEURONS CONTROL POLLUTION-MEDIATED NEUTROPHILIC ASTHMA

**DOI:** 10.1101/2024.08.22.609202

**Published:** 2024-08-23

**Authors:** Jo-Chiao Wang, Theo Crosson, Amin Reza Nikpoor, Surbhi Gupta, Moutih Rafei, Sebastien Talbot

**Affiliations:** 1 Department of Pharmacology and Physiology, University de Montreal, Canada; 2 Department of Physiology and Pharmacology, Karolinska Institutet, Sweden; 3 Department of Biomedical and Molecular Sciences, Queen’s University, Canada

## Abstract

The immune and sensory nervous systems, having evolved together, use a shared language of receptors and transmitters to maintain homeostasis by responding to external and internal disruptions. Although beneficial in many cases, neurons can exacerbate inflammation during allergic reactions, such as asthma. Our research modeled asthma aggravated by pollution, exposing mice to ambient PM_2.5_ particles and ovalbumin. This exposure significantly increased bronchoalveolar lavage fluid neutrophils and γδ T cells compared to exposure to ovalbumin alone. We normalized airway inflammation and lung neutrophil levels by silencing nociceptor neurons at inflammation’s peak using intranasal QX-314 or ablating TRPV1-expressing neurons. Additionally, we observed heightened sensitivity in chemical-sensing TRPA1 channels in neurons from pollution-exacerbated asthmatic mice. Elevated levels of artemin were detected in the bronchoalveolar lavage fluid from pollution-exposed mice, with artemin levels normalizing in mice with ablated nociceptor neurons. Upon exposure PM_2.5_ particles, alveolar macrophages expressing pollution-sensing aryl hydrocarbon receptors, were identified as the source of artemin. This molecule enhanced TRPA1 responsiveness and increased neutrophil influx, providing a novel mechanism by which lung-innervating neurons respond to air pollution and suggesting a potential therapeutic target for controlling neutrophilic airway inflammation in asthma, a clinically intractable condition.

## INTRODUCTION.

Wildfire events and severity have increased significantly, with substantial adverse health impacts from wildfire-attributable (particulate matter) PM_2.5_ exposure. The mortality burden from PM_2.5_ exposure due to California fires from 2008 to 2018 was evaluated to ~53,000 premature deaths over the 11 years, resulting in an economic impact of $432 to $450 billion^1^. This form of asthma characterized by significant neutrophilic infiltration is often resistant to traditional treatments, highlighting a significant unmet need in respiratory healthcare^2^. Recent estimates suggest that neutrophilic asthma accounts for approximately 15–25% of all asthma cases, with around 50% of these cases being refractory to standard asthma treatments^3^. Taken together, resolving neutrophilic asthma constitutes an unmet clinical need of increasing importance.

Organisms have evolved complex fail-safe systems to maintain homeostasis, integrating mechanisms for threat detection, reflex responses, and tailored immune responses^4^. These functions are co-orchestrated by the immune and sensory nervous systems and designed to detect external threats and internal disturbances. To achieve this, both systems utilize shared metabolic pathways and a common signaling repertoire, including receptors, cytokines, and neuropeptides^5^. This shared molecular framework facilitates continuous interactions between these systems, which is crucial for regulating various processes. These include the physiological monitoring of barrier tissues such as skin^6^, lung^7^, and gut^8^ and involve anticipatory immune responses^9^ as well as the management of pathologies like allergic reactions^10,11^, infections^12^, and malignancies^13–16^.

In lung disease, vasoactive intestinal peptide **(VIP**), released from a subset of pulmonary sensory neurons stimulated by the inflammatory cytokine IL-5, promotes allergic inflammation. It acts on CD4^+^ T cells and resident innate lymphoid type 2 cells (**ILC2**s), increasing T helper type-2 (**T**_**H**_**2**) cytokines, which are key drivers of asthmatic conditions^17,18^. Building on these findings, our research indicates that vagal nociceptor neurons contribute to airway hyperresponsiveness^4,19^, mucus metaplasia^20,21^, and sensing of immunoglobulin E and G^22,23^. These neurons also direct antibody class switching in B cells^24^ and IgG production^25^. Our recent studies have revealed that a subset of nociceptor neurons is actively reprogrammed by asthma-driving cytokines IL-4/IL-13, making them sensitive to the inhibitory signal provided by NPY secreted by lung-innervating sympathetic neurons^26^. Additionally, we have discovered that lung-resident basophils sustain the pro-inflammatory phenotype of a population of vagal preceptors^27^. Other work shows that leukotriene C4 and IL-4 receptors, namely CysLTR2 and IL-4Rα, are associated with chronic itching and exacerbated bronchial contraction and inflammation in asthma^28^.

Urban pollution and the increasing frequency and severity of wildfires contribute to higher concentrations of small particulate matter (less than 2.5 μm). This change shifts asthma from treatment-responsive eosinophilic and T_H_2 types to treatment-resistant neutrophilic and mixed T_H_17/T_H_2 types. To address this unmet clinical need and explore whether neuron silencing can alleviate this type of airway inflammation, we used a novel model of neutrophilic asthma. In this model, fine particulate matter (**FPM**) is added to ovalbumin. We also investigated the molecular mechanisms behind the heightened neuronal sensitivity observed in this context, identifying alveolar macrophages-produced artemin as a key driver of this hypersensitivity.

## RESULTS.

The group led by Prof. Ya-Jen Chang^29^ has recently developed a novel method to model pollution-exacerbated asthma and to explore how fine particulate matter (**FPM**) causes airway inflammation. Their research demonstrates that FPM exposure triggers airway hyperreactivity (**AHR**) and neutrophilic inflammation, increases T_H_1 and T_H_17 immune responses, and elevates epithelial cell apoptosis rates. A key finding is that γδ T cells significantly contribute to this inflammation and AHR by producing IL-17A.

**Figure 1.**
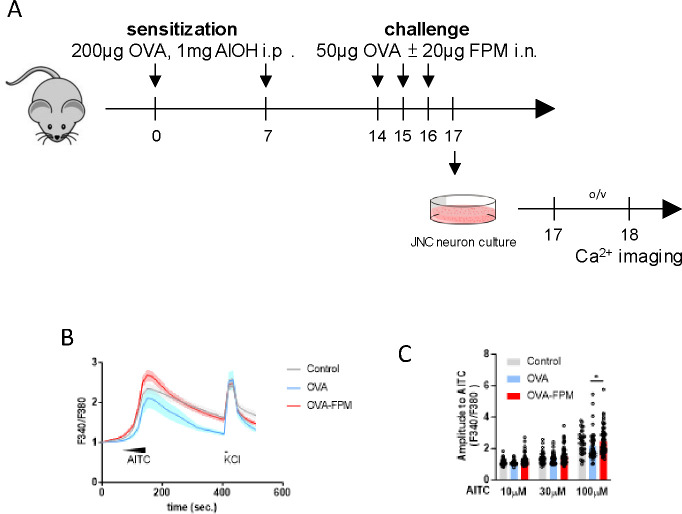
Air pollution exacerbates nociceptor neurons’ activity. (**A-C**) Male and female C57BL6 mice aged 6–10 weeks were sensitized via intraperitoneal injection with an emulsion containing ovalbumin (OVA, 200 μg/dose) and aluminum hydroxide (1 mg/dose) on days 0 and 7. They were then challenged intranasally with OVA (50 μg/dose) with or without fine particulate matter (FPM, 20 μg/dose) on days 14–16. Bronchoalveolar lavage fluid (BALF) was collected, and JNC neurons were cultured on day 17. (**B-C**) JNC neurons were harvested and cultured for 24 hours. Following culturing, the cells were loaded with the calcium indicator Fura-2AM and sequentially stimulated with AITC (10 μM at 60–90 seconds, 30 μM at 90–120 seconds, 100 μM at 120–150 seconds) and KCl (40 mM at 420–435 seconds). Calcium flux was recorded throughout these stimulations (**B**), and the amplitude of AITC responses was calculated as the ratio of peak F340/F380 after stimulation to the baseline F340/F380 30 seconds before stimulation (**C**). Data are presented as means ± 95% CI (**B**), means ± SEM (**C**) (**B-C**) Representative data from 2 independent experiments. AITC-responsive neurons were included in amplitude analysis. n=35 neurons from control mice, n=42 neurons from OVA-treated mice, and n=76 neurons from OVA-FPM-treated mice were analyzed. Statistical significance is indicated by *p≤0.05, **p≤0.01, ***p≤0.001, ****p≤0.0001.

Building on this foundation, our team is investigating whether lung-innervating nociceptor neurons become sensitized during asthma exacerbations. To test this, male and female C57BL6 mice aged 6–10 weeks were sensitized with an emulsion of ovalbumin (OVA, 200 μg/dose) and aluminum hydroxide (1 mg/dose) on days 0 and 7 and later challenged intranasally with OVA (50 μg/dose) with or without fine particulate matter (FPM, 20 μg/dose) on days 14–16. JNC neurons were harvested on day 17, cultured for 24 hours, and then loaded with the calcium indicator Fura-2AM (Fig. 1A). Subsequent stimulation with the TRPA1 agonist AITC (10 μM) and the pan-neuronal activator KCl (40 mM) revealed increased neuronal responsiveness in mice exposed to the FPM and OVA combination compared to those exposed only to OVA. The latter showed similar sensitivity to the one of wild-type cells (Fig. 1B–C).

**Figure 2.**
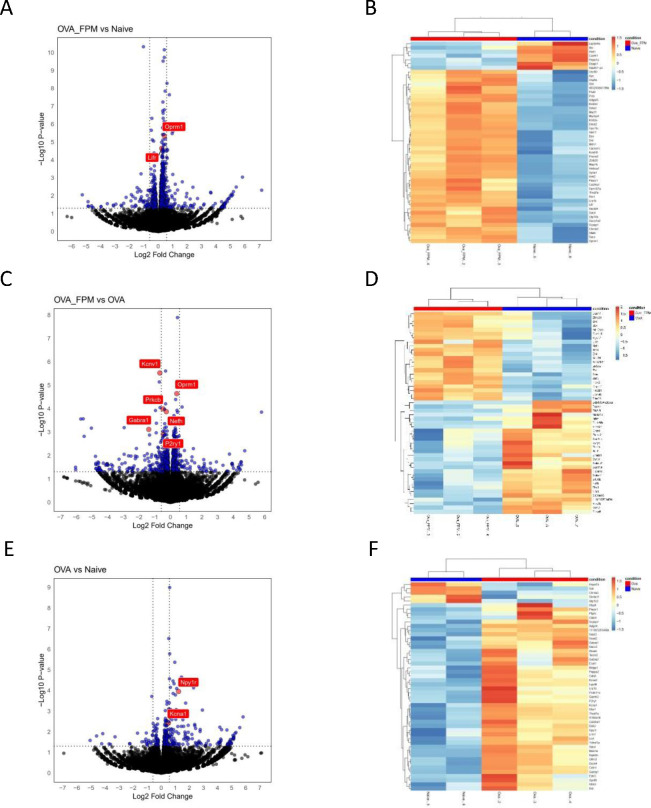
Air pollution reprogram nociceptor neurons’ transcriptome. (**A-F**) Naïve male and female TRPV1^cre^::td-Tomato^cre/wt^ mice, aged 6–10 weeks, were subjected to either a pollution-exacerbated asthma protocol, the classic ovalbumin (OVA) protocol, or were kept naïve. On day 17, at the peak of inflammation, JNC neurons were harvested, dissociated, TRPV1^+^ neurons FACS-purified (td-tomato+) from stromal cells and non-peptidergic neurons, and isolated for RNA sequencing. Differentially expressed genes were calculated and displayed as volcano plots and heatmaps for the comparisons: OVA-FPM vs. naïve (**A-B**), OVA-FPM vs. OVA (**C-D**), OVA alone vs. naïve (**E-F**). Significant neuronal reprogramming was observed and characterized by the overexpression in the OVA-FPM vs. naïve groups (Lifr, Oprm3), OVA-FPM vs. OVA alone groups (Oprm1, Nefh, P2ry1, Prkcb, Gabra1, Kcnv1), and OVA alone vs. naïve groups (Npy1r, Kcna1). Data are presented as volcano plot (p < 0.05; **A, C, E**) or heatmap (**B, D, F**) showing normalized gene expression (log_2_ (0.01 + transcripts per million reads (TPM)) – mean). The sequencing experiment was not repeated. n consisted of 2–3 mice.

Building on recent findings that the asthma-promoting cytokines IL-4 and IL-13 reprogram lung-innervating nociceptor neurons to adopt a pro-allergic phenotype^26^, we sought to understand how pollution-exacerbated asthma might reprogram these neurons. To do so, TRPV1^cre^::td-Tomato^cre/wt^ mice underwent the classic ovalbumin protocol with or without FPM exposure. TRPV1^+^ JNC neurons were isolated, dissociated, and enriched to exclude adjacent satellite glial and immune cells, followed by FACS-sorting and RNA sequencing. Our data suggest the induction of several differentially expressed genes (DEGs) between OVA-FPM vs naïve groups (*Lifr, Oprm1*) (Fig. 2A–B), OVA-FPM vs OVA alone groups (*Oprm1, Nefh, P2ry1, Prkcb, Gabra1, Kcnv1*) (Fig. 2C–D), and OVA alone vs naïve groups (*Npy1r, Kcna1*) (Fig. 2E–F). Together, the induction of pollution-exacerbated asthma transcriptomically and functionally reprogram lung-innervating nociceptor neurons.

Building on this finding, we explore whether silencing these neurons affects pollution-exacerbated asthma. To test this, we adapted a neuron-blocking strategy, originally developed for pain and itch neurons, to silence tumor-innervating nociceptors. This approach utilizes non-selective ion channels (TRPA1 and TRPV1) as drug-entry ports for delivering a charged, membrane-impermeable form of lidocaine (QX-314). QX-314 enters sensory fibers during inflammation to block sodium currents, providing a targeted and durable (> 9h) electrical blockade of nociceptors without affecting immune cell function^30–33^. Intranasal injection of QX-314, a charged calcium channel blocker reversed allergic airway inflammation, coughing, mucus metaplasia, and hyperreactivity^18^. Here, we found that an acute intranasal dose of QX-314 at the peak of inflammation (day 17, 5 nmol, 50 μL) significantly reduced bronchoalveolar lavage fluid (BALF) eosinophil and neutrophil counts, normalizing these levels to the one observed in asthmatic mice (Fig. 2A–B). To substantiate these findings, we genetically engineered mice with either intact (TRPV1^wt^::DTA^fl/wt^) or ablated (TRPV1^cre^::DTA^fl/wt^) TRPV1-expressing nociceptor neurons^13,34,35^. Compared to controls, the ablated mice showed a marked reduction in BALF neutrophils and γδ T cells (Fig. 2C–E), confirming the significant role of nociceptors in modulating inflammation and immune responses in pollution-exacerbated asthma.

To further understand how nociceptor neurons regulate airway inflammation, we conducted an unbiased multiplex cytokine array. This analysis revealed elevated levels of asthma-driving cytokines IL-4 and IL-13, as well as the pro-inflammatory cytokines IL-6, CCL2, and TNFα, with TNFα levels normalizing following the ablation of nociceptor neurons (Fig. 2F). Additionally, targeted ELISA analysis showed increased levels of artemin, which returned to normal in the absence of nociceptor neurons (Fig. 2G). Artemin is a significant protein within the glial cell line-derived neurotrophic factor (GDNF) family, which is crucial for the development and functionality of the nervous system. Artemin is vital in developing sympathetic^36^ and sensory neurons^37^ by binding to the GFRα3-RET receptor complex on their surfaces, including many nociceptors. Activation of this receptor by artemin not only supports the survival and growth of these neurons during embryonic development but also leads to their increased sensitivity and hyperactivity under inflammatory conditions^38–40^.

**Figure 3.**
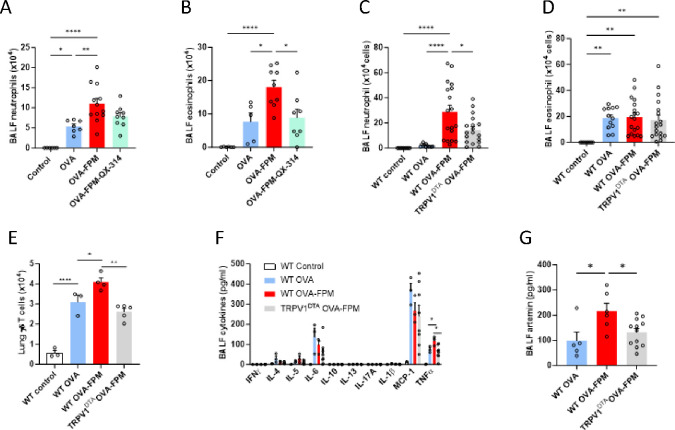
Nociceptor neurons control pollution-exacerbated asthma. (**A-B**) Male and female C57BL6 mice aged 6–10 weeks were sensitized via intraperitoneal injection with an emulsion containing ovalbumin (200 μg/dose in 100 μl) and aluminum hydroxide (1 mg/dose in 100 μl) on days 0 and 7. They were then challenged intranasally with OVA (50 μg/dose in 50 μl) with or without fine particulate matter (FPM, 20 μg/dose in 50 μl) on days 14–16 and received intranasal injections of QX-314 (5 nmol/dose in 50 μl) on day 16, 30 minutes after the last challenge. Bronchoalveolar lavage fluid (BALF) was collected on day 17 and analyzed immune influx immunophenotyped by flow cytometry. Compared to naïve mice or OVA-exposed mice, the one co-challenged with OVA-FPM showed increased BALF infiltration of neutrophils (**A**) and eosinophils **(B**). The mice treated with QX-314 were protected from these increases (**A-B**). (**C-G**) Male and female littermate control (TRPV1^wt^::DTA^fl/wt^) and nociceptor ablated (TRPV1^cre^::DTA^fl/wt^) mice aged 6–10 weeks were sensitized via intraperitoneal injection with an emulsion containing ovalbumin (OVA, 200 μg/dose) and aluminum hydroxide (1 mg/dose) on days 0 and 7. They were then challenged intranasally with OVA (50 μg/dose) with or without fine particulate matter (FPM, 20 μg/dose) on days 14–16. Bronchoalveolar lavage fluid (BALF) was collected on day 17, and immune influx was immunophenotyped by flow cytometry. Compared to naïve mice or OVA-exposed mice, the one co-challenged with OVA-FPM showed increased BALF infiltration of neutrophils (**C**) and γδ T-cells (**E**). The nociceptor ablated (TRPV1^cre^::DTA^fl/wt^) mice were protected from these increases (**C, E**) but showed similar levels of BALF eosinophils (**D**). (**F-G**) BALF cytokines were measured using a cytokine multiplex array and ELISA, and increased levels of IL-5, MCP1 (CCL2), TNFα, and artemin were found. TNFα (**F**) and artemin (**G**) rise were absent in nociceptor-ablated mice. (**A-G**) Data are presented as means ± SEM. (**A-B**) Pooled data from 2 independent experiments. n=6–12 mice per group. (**C-E**) Pooled data from 2 independent experiments. n=9–18 mice per group. (**F**) Representative data from 2 independent experiments. n=3–6 mice per group. (**G**) Representative data from 2 independent experiments. n=2–8 mice per group. (**J**) Pooled data from 2 independent experiments. n=5–12 mice per group. Statistical significance is indicated by *p≤0.05, **p≤0.01, ***p≤0.001, ****p≤0.0001.

To better understand the role of artemin and its interactions in respiratory health, we focused on identifying the specific populations of vagal neurons that express the artemin receptor, Gfrα3. To achieve this, we conducted an in-silico analysis of single-cell RNA sequencing data from the jugular-nodose ganglion cells of Kupari (dataset GSE124312)^41^. This analysis revealed that *Gfra3* is expressed in several nociceptor neuron subtypes (Fig. 3A–B), specifically JG3 (OSM-R-expressing neurons), JG4 (TRPA1 and SSTR2-expressing), and JG6 (TRPM8-expressing).

In parallel, we explored the cellular sources of artemin and the aryl hydrocarbon receptor (AhR) using Immgen. Our analysis indicated high expression of the artemin gene (*Artn*) by macrophages, showing significant levels of AhR expression. However, slightly less than ILC2s and eosinophils (SF. 1). Based on these in silico findings, we harvested alveolar macrophages from naïve C57BL/6 mice and exposed them to fine particulate matter (FPM). This resulted in time-dependent increases in *Artn* transcript expression, underscoring the dynamic response of these cells to environmental stimuli (Fig. 3C–D). These findings elucidate significant cellular interactions involving artemin and AhR, highlighting their roles in inflammation and potentially in the pathophysiology of respiratory diseases.

A recent study has linked the overexpression of artemin with increased mRNA levels of several key nociceptor neurons inflammation markers including *Gfrα3, TrkA, Trpv1,* and *Trpa1*, which was associated with the enhancement of thermal sensitivity in mice by lowering heat thresholds and amplifying responses to thermal stimuli^39^. Building on this, our investigations into FPM-sensitized neurons revealed heightened calcium responses to the TRPA1 agonist AITC (Fig 1B–C), suggesting a potential role for artemin in this enhanced activity. Subsequent experiments conducted with naïve C57BL/6 mice provided further evidence; JNC neurons from these mice demonstrated increased AITC responses compared to those treated with a vehicle (Fig 1F–H). These findings collectively suggest that alveolar macrophages, by releasing artemin, act as an upstream mediator sensitive to pollutants. This mechanism leads to sensitizing nociceptor neurons, facilitating their role in upregulating allergic airway inflammation. This pathway underscores a critical interaction between environmental pollutants and the neurogenic exacerbation of allergic responses (SF 2).

## DISCUSSION.

Our research, along with others, indicates that nociceptor neurons typically promote regulatory immunity in the context of bacterial^42^, viral^43^, fungal^44^ infections, or malignancies^13^. To our knowledge, the impact of neuro-immunity in regulating T_H_17 immunity is more limited, with only one study showing that TRPV1^+^ neuron activation elicits a local type 17 immune response that augmented host defense to C. albicans and S. aureus^9^. Given the knowledge gap in lung inflammation, we set out to assess how neuro-immunity shapes neutrophil influx and T_H_17/T_H_2 activation in a model of pollution-exacerbated airway inflammation. We found that ablation or silencing of nociceptor neurons prevented the induction of airway inflammation, highlighting a potential novel therapeutic pathway for treating refractory asthma. This finding phenocopy our previous preclinical data showing that nociceptor neuron silencing using a charged voltage-gated sodium channel^18,24,30^ or calcium channel^31^ blocker can stop eosinophilic airway inflammation. It also matches the absence of effect we found in nociceptor neuron ablation in a model of T_H_1 inflammation induced by CFA/OVA^17^.

### Dissecting lung neuro-immunity.

Chiu’s group demonstrated that during bacterial infections, nociceptor neurons limit neutrophil influx and their antimicrobial activity by releasing the neuropeptide CGRP^12^. In support of this, Ugolini’s team found that ablating Na_V_1.8^+^ nociceptor neurons during HSV-1 viral infections led to a significant increase in neutrophils in the skin, associated with heightened cytokine production and viral skin lesions^43^. Here, we report that ablating TRPV1 neurons, a subset of Na_V_1.8-expressing sensory neurons, decreases the influx of neutrophils exacerbated by pollution, suggesting a different underlying mechanism, likely involving substance P rather than CGRP.

Thus, we showed that vagal nociceptor neurons can detect immune complexes formed between allergens and IgE, triggering the release of substance P (SP) and vasoactive intestinal peptide (VIP) but not CGRP^22^. We confirmed these findings in asthmatic mice’s bronchoalveolar lavage fluid (BALF)^24^. Further, we demonstrated that SP promotes mucus metaplasia in the lungs of asthmatic mice^20^ and influences B cell antibody class switching^24^. Other researchers corroborated both findings^25,45–47^. Additionally, we observed that TRPV1^+^ nociceptor neuron ablation impairs antigen trafficking in lymph nodes^48^, and antibody class-switching^25^ potentially reducing the severity or development of allergic reactions^49^.

Emerging research highlights the varied immunomodulatory effects of different neuropeptides. For instance, CGRP hinders the migration of dendritic cells in psoriasis^11,50^, whereas SP enhances their migration to lymph nodes in atopic dermatitis^10^. VIP and neuromedin U (NMU) increase the production of pro-asthmatic cytokines by lung ILC2 cells^18,51–54^, whereas CGRP has similar^55,56^ or opposite effect^57^. In the lungs, CGRP reduces the infiltration of neutrophils and gamma-delta T (γδT) cells, offering protection against Staphylococcus aureus pneumonia^58,59^. Conversely, CGRP can worsen psoriasis in the skin by inducing dendritic cells to produce IL-23, which activates IL-17-producing γδT cells, exacerbating inflammation^44^. In response to C. albicans, sensory neurons also release CGRP, prompting CD301b^+^ dendritic cells to produce IL-23 and initiating a T helper type-17 (T_H_17) and γδT cell-mediated response marked by IL-17A and IL-22 production, which enhances host resistance to infection^9^. Our findings indicate that nociceptor neuron ablation reduces γδ T-cell activation, an effect we attribute to SP/VIP-driven responses due to our model’s lack of heighten CGRP release^18,24^. Future research will focus on how neurons directly regulate γδ T-cell and neutrophil function in these contexts.

### Aryl hydrocarbon receptor and alveolar macrophages

The aryl hydrocarbon receptor (AhR) is essential in modulating inflammation, as evidenced by its ability to decrease inflammation in the skin of psoriasis patients^60^. Mice lacking AhR display worsened symptoms, underscoring its vital role in controlling inflammation^61^. Beyond its expression in alveolar macrophages, as we observed, AhR is also present in various other innate immune cells such as ILC2, eosinophils, and neurons. A recent preprint highlights AhR’s dual function as a critical molecular sensor and regulator, balancing neural protection and axon regeneration^62^. It shows that while AhR activation in DRG sensory neurons inhibits axon regeneration, its deletion activates gene programs associated with axonogenesis, reduces inflammation and stress signaling, and enhances pro-growth pathways following peripheral axotomy^62^. AhR has also been implicated in regulating the gut-brain axis^63^. Although we have not formally tested this, it is plausible that AhR-expressing neurons, similar to alveolar macrophages, can directly sense fine particulate matter (FPM), potentially explaining the direct neuronal reprogramming we observed after pollutant exposure. Future research will explore this hypothesis using nociceptor neurons with conditional AhR knockout.

### Artemin sensing, TRPA1 sensitization, and airway inflammation.

Alveolar macrophages serve as an early warning system in the lungs^64^. We found that they detect pollutants like fine particulate matter and trigger defensive reflexes by releasing artemin. This action activates and sensitizes nociceptor neurons to TRPA1-sensing noxious stimuli. Single-cell RNA sequencing revealed that a subtype of TRPA1, SST2R neurons, express the GDNF receptor GFRα3 and are sensitized by artemin. This finding complements previous research showing that keratinocyte-produced TSLP sensitizes skin-innervating nociceptor neurons, promoting itch and skin atopy^65^. In the lungs, TRPA1 germline knockout reduces allergic airway inflammation^66,67^, while Genentech’s recent Phase 1 research demonstrated that TRPA1 agonists are elevated in asthmatic human airways and contribute to inflammation and hyperreactivity. Their development of GDC-0334, a selective TRPA1 antagonist, effectively reduces inflammation, cough, and allergic reactions in preclinical trials and decreases pain and itch in human studies^68^, supporting TRPA1 sensitization and nociceptor neuron as a whole as a major upstream asthma driver.

We previously observed that neurons in the nodose ganglia express high levels of TRPA1 and exhibit increased thermal sensitivity and neuron outgrowth in response to brain-derived neurotrophic factor (BDNF) yet remain unresponsive to nerve growth factor^35^. Here, we showed that lung-innervating vagal neurons express GFRa3 and respond to artemin, which sensitizes TRPA1 responses and promotes subsequent airway inflammation. JNC neurons show significant transcriptomic reprogramming when exposed to ovalbumin plus fine particulate matter (OVA+FPM), like their response to pro-asthmatic cytokines. Previous studies support this reprogramming and TRPA1 sensitization. Among others, data shows that EGR1 and artemin levels are elevated in atopic dermatitis patients, where EGR1-deficient mice showed reduced nerve density and scratching behavior mediated by artemin signaling^69^. Overexpression of artemin also increases the sensitivity of sensory neurons to thermal stimuli^39^, highlighting its crucial role in regulating neuronal outgrowth and sensitivity in atopic conditions. Taking together, preventing artemin’s action on neurons by blocking GFRa3 might also reveal novel therapeutic targets to avoid the maladaptive involvement of nociceptor neurons in pollution-exacerbated asthma.

In the context of pollution-exacerbated asthma, our research links the sensitization of TRPA1^+^ nociceptor neurons and heightened allergic inflammation to artemin produced by macrophages. We propose new therapeutic targets to dampen neutrophilic airway inflammation by i) targeting the AhR-mediated pollution sensing by alveolar macrophages (AM), ii) inhibiting artemin’s effect on neurons by blocking GFRa3, iii) blocking TRPA1 using novel antagonists such as GDC-0334, and iv) silencing nociceptor neurons with charged lidocaine derivatives.

## MATERIALS AND METHODS

### Animals and in vivo experiments

All procedures involving animals adhered to the guidelines of the Canadian Council on Animal Care (CCAC) and the Queen’s University Animal Care Committee (UACC). Mice were accommodated in individually ventilated cages with free water and food access under 12-hour light cycles. The strains used, C57BL6/J (000664), DTA^fl/fl^ (010527), and TRPV1^cre^ (017769), were obtained from the Jackson Laboratory and bred in-house.

For the ovalbumin (OVA)-induced allergic airway inflammation model, C57BL6 mice were sensitized via intraperitoneal injection with a mix of grade V OVA (200 μg/dose; Sigma-Aldrich A5503) and Imject^®^ Alum (1 mg/dose; ThermoFisher 77161) on days 0 and 7. Subsequently, they underwent intranasal challenges with OVA (50 μg/dose), with or without fine particulate matter (FPM; 20 μg/dose; NIST 2786), from day 14 to 16. Control mice were sensitized but not challenged. The mice were euthanized on day 17, and samples from bronchoalveolar lavage fluid, lung tissue, and the jugular-nodose complex (JNC) were collected.

### Bronchoalveolar lavage

Bronchoalveolar lavage was performed on mice anesthetized using previously described tracheal incision methods. The mice underwent lavage twice with 1 ml of either PBS or FACS buffer (2% FBS and one mM EDTA in PBS) utilizing a Surflo ETFE IV Catheter 20G × 1” (Terumo Medical Products SR-OX2025CA). The lavage fluid was then centrifuged at 350 × G for 6.5 minutes. The supernatant was collected for ELISA analysis, while the cell pellets were resuspended, treated with RBC lysis solutions (Cytek TNB-4300-L100 or Gibco A1049201), and stained for surface markers for subsequent flow cytometry analysis.

Lung tissues were harvested after the diaphragm incision, and transcardial perfusion was performed with 10 ml of PBS. The tissues were then finely minced using razor blades and collected either into TRIzol^™^ Reagent (Invitrogen 15596026) for RNA extraction or into a digestion buffer containing 1.6 mg/ml collagenase type 4 (Worthington LS004189) and 100 μg/ml DNase I (Roche 11284932001) in supplemented DMEM for the preparation of a single-cell suspension. This suspension was achieved through 45 minutes of enzymatic digestion at 37°C. Mechanical dissociation was carried out using 18-gauge needles halfway through the digestion period (30 minutes), then straining through a 70 μm nylon mesh and RBC lysis. For flow cytometry or fluorescence-activated cell sorting (FACS), the cells were resuspended in FACS buffer; for in vitro stimulation, the cells were resuspended in FBS-supplemented DMEM, seeded into 96-well plates and cultured at 37°C with 5% CO2 for the designated time before collecting the supernatant.

### Alveolar macrophage culture

Alveolar macrophages were isolated from the bronchoalveolar lavage fluid (BALF) of naïve mice, with these cells constituting approximately 95% of the sample. The cells were centrifuged and treated with RBC lysis as previously described, then cultured overnight at a density of 3 × 10^5 cells per well in 96-well plates. The culture medium used was DMEM (Gibco 11965092) supplemented with one mM sodium pyruvate (Gibco 11360070), two mM GlutaMAX^™^ (Gibco 35050061), 100 U/mL penicillin and 100 μg/mL streptomycin (Corning 30–002-CI), ten mM HEPES (Gibco 15630080), and 10% FB Essence (VWR 10805–184).

The cells were then stimulated with 100 μg/ml fine particulate matter (FPM; NIST 2786) for 1–4 hours. Following stimulation, RNA was extracted for quantitative PCR (qPCR) analysis.

### Neuron culture

The jugular-nodose complex (JNC) was collected after exsanguinating anesthetized mice. The ganglia were placed into a digestion buffer composed of 1 mg/ml (325 U/ml) collagenase type 4 (Worthington LS004189), 2 mg/ml (1.8 U/ml) Dispase II (Sigma 04942078001), and 250 μg/ml (735.25 U/ml) DNase I (Roche 11284932001), prepared in supplemented DMEM media without FB Essence. This mixture was incubated at 37°C for 60 minutes to facilitate enzymatic digestion. Mechanical dissociation was then performed by repeatedly pipetting the digested tissue with pipette tips of decreasing diameter, concluding with 25-gauge needles. This was followed by density gradient centrifugation at 200 g for 20 minutes, with low acceleration and deceleration settings, using a layer of 150 mg/ml bovine serum albumin (BSA; Hyclone SH30574.02) in a PBS solution.

The cells at the bottom were collected, RBC lysed, and then seeded onto glass-bottom dishes (ibidi 81218) pre-coated with 50 μg/ml laminin (Sigma L2020) and 100 μg/ml poly-D-lysine (Sigma P6407). They were cultured overnight in Neurobasal-A media (Gibco 10888022) supplemented with one mM sodium pyruvate (Gibco 11360070), two mM GlutaMAX^™^ (Gibco 35050061), 100 U/mL penicillin, 100 μg/mL streptomycin (Corning 30–002-CI), ten mM HEPES (Gibco 15630080), B-27 supplement (Gibco 17504–044), 50 ng/ml mouse nerve growth factor (NGF; Gibco 13257–019), two ng/ml mouse glial-derived neurotrophic factor (GDNF; Novus NBP2–61336), and cytosine-β-D-arabinofuranose (Thermo Scientific J6567106). In some experiment, 100 ng/ml artemin or 50 M HCl (as vehicle control) were treated *in vitro* to dishes, without NGF or GDNF supplements. This setup was used for subsequent calcium imaging recordings.

### Calcium imaging recording

Cultured neurons were loaded with the calcium indicator dye, five μM fura-2 AM (Cayman Chemical Company 34993), and incubated at 37°C for 40 minutes. Following incubation, the neurons were washed four times with standard external solution (SES; Boston BioProducts C-3030F) before imaging in the same solution. The data captured from the Fura-2 signals were utilized for further analysis. Agonists, diluted in SES, were administered using a ValveLink8.2 system (AutomateScientific) equipped with 250 μm Perfusion Pencil^®^ tips (Automate Scientific) and controlled via Macro Recorder (Barbells Media, Germany). To ensure complete drug washout, SES continuously flowed between drug injections.

Imaging for Fura-2 experiments was performed using an S Plan Fluor ELWD 20X objective lens (NIKON) to enhance UV light transmission. In contrast, GcaMP6f experiments employed an S Plan Fluor LWD 20X lens (NIKON) to achieve improved resolution. Images were captured every 3 or 4 seconds using sCMOS cameras, including pco. Edge 4.2 LT (Excelitas Technologies), Prime 95B (Teledyne Photometrics), and Orca Flash 4.0 v2 (Hamamatsu Photonics). All imaging activities were conducted on ECLIPSE Ti2 Inverted Microscopes (NIKON).

Regions of interest (ROI) were manually delineated using NIS-Elements software (NIKON), and the F340/F380 ratios were exported to Excel (Microsoft) for further analysis. Data were condensed into a maximum value every 15 seconds for all analyses.

### Flow cytometry

Single-cell suspensions derived from bronchoalveolar lavage fluid (BALF) or lung samples were stained with Ghost Dye Violet 510 (Cytek, 13–0870-T100) and antibody cocktails in PBS. The cells were incubated at four °C for 30 minutes, followed by fixation with 10% neutral buffered formalin (Sigma Aldrich, HT501128) at room temperature for 15 minutes before acquiring flow cytometry data. For the assessment of eosinophil and neutrophil infiltration, BALF cells were stained with fluorochrome-conjugated antibodies targeting CD45 (30-F11), CD90.2 (53–2.1), CD11b (M1/70), CD11c (N418), Ly6C (HK1.4), Ly6G (1A8), and Siglec-F (1RNM44N). For the analysis of γδ T cells in lung cells, the staining involved CD45 (30-F11), TCRγδ (GL3), CD90.2 (53–2.1), and lineage markers including TCRβ (H57–597), CD19 (1D3/CD19), NK1.1 (PK136), CD11b (M1/70), CD11c (N418), F4/80 (BM8), and FcεRIα (MAR-1), sourced from Biolegend or Thermo Fisher Scientific. Data was acquired using a FACS Canto II (BD Biosciences).

### Real-time quantitative PCR (qPCR)

Stimulated alveolar macrophages were lysed using TRIzol Reagent and stored at −80°C before RNA extraction. RNA from sorted cells was extracted using the PureLink RNA Micro Scale Kits (ThermoFisher, 12183016), while RNA from lung tissues or lung cell suspensions was extracted with the E.Z.N.A.^®^ Total RNA Kit I (Omega Bio-tek^®^, R6834). All RNA extractions followed the manufacturer’s instructions, including phenol-chloroform phase-based purification and mixing with equal volumes of isopropanol.

cDNA synthesis was conducted using SuperScript VILO Master Mix (Invitrogen, 11755050), utilizing 1–2 μg of RNA as a template for each reaction. Quantitative polymerase chain reaction (qPCR) was carried out with PowerUp SYBR Green Master Mix (Applied Biosystems, A25742), using 50–100 ng of cDNA templates and 200 nM of respective primers. The qPCR was executed on a Mic qPCR Cycler (Bio Molecular Systems) or a CFX Opus Real-Time PCR System (Bio-Rad Laboratories). The primer pair used for the *Artn* gene was forward TGATCCACTTGAGCTTCGGG and reverse CTCCATACCAAAGGGGTCCTG.

### Enzyme-linked immunosorbent assay (ELISA)

artemin in BALF is determined by ELISA (R&D system DY1085–05) according to the manufacturer’s manual. Inflammatory cytokines in BALF were detected by Cytometric Beads Array Flex Sets purchased from BD Biosciences: Master Buffer Set (558266), IL-1β (560232), IL-4 (558298), IL-5 (558302), IL-6 (558301), IL-10 (558300), IL-13 (558349), IL-17A (560283), IFNγ (558296), MCP-1 (558342), and TNF (558299) according to the manufacturer’s manual.

### *In silico* analysis of RNA-Seq data

Data were extracted from Kupari et al.’s supplementary materials, with clusters defined according to the original publication^41^. Bulk JNC sequencing was analyzed with DESeq2. Immgen data were extracted as csv and plotted with GraphPad Prism. Data from single-cell portal was extracted and plotted with R.

### Data availability

Information and raw data are available from the lead contact upon reasonable request. (GEO pending)

### Statistics

P *values* ≤ *0.05* were considered statistically significant. One-way ANOVA, two-way ANOVA, and Student t-tests were performed using GraphPad Prism. DESeq2 and Seurat analysis and statistics were performed using RStudio.

### Replicates

Replicates (n) are described in the figure legends and represent the number of animals for *in vivo* data. For *in vitro* data, replicates can either be culture wells or dishes, animals, fields-of-view (microscopy), or neurons (calcium microscopy), but always include different preparations from different animals to ensure biological reproducibility.

## Supplementary Material

Supplement 1

## Figures and Tables

**Figure 4.**
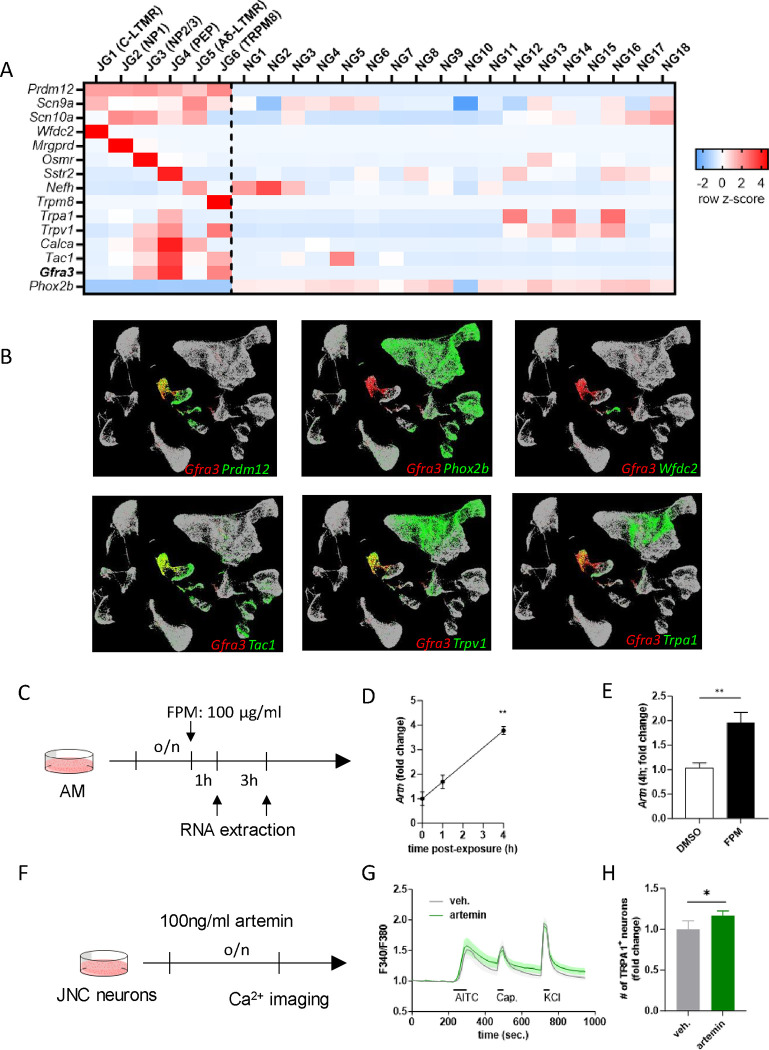
Artemin sensitizes TRPA1 activity in vagal sensory neurons. (**A**) In-silico analysis of the dataset GSE124312 generated a heatmap showing the transcript expression levels of the pan neural-crest lineage transcription factor (*Prdm12*), voltage-gated sodium channels (*Scn9a, Scn10a*), jugular subset markers (*Wfdc2, Mrgprd, Osmr, Sstr2, Nefh, Trpm8*), peptidergic neuron markers (*Trpa1, Trpv1, Calca, Tac1, Gfra3*), and the pan placodal lineage marker (Phox2b). Experimental details and cell clustering are defined in Kupari et al.^41^. (**B**) In-silico analysis of GSE192987 presenting the co-expression of Gfra3 with TRPA1 or other inflammatory markers. The data are presented as a heatmap displaying the row z-scores (A) or cells that has TPTT>1 in UMAPs. (**C-E**) Alveolar macrophages from naïve male and female C57BL6 mice (3 × 10^5 cells/well) were cultured overnight and stimulated with DMSO or FPM (100 μg/ml). RNA was extracted at 1 and 4h post-stimulation, and *Artn* expression was analyzed using qPCR (**C**). Data showed that FPM increased artemin transcript expression 1 and 4h post-stimulation (**D-E**). (**F-H**) JNC neurons were harvested, pooled, and cultured overnight with either vehicle or artemin (100 ng/mL). The cells were then sequentially stimulated with AITC (TRPA1 agonist; 300 μM at 240–270 seconds), capsaicin (TRPV1 agonist; 300 nM at 320–335 seconds), and KCl (40 mM at 720–735 seconds). The percentage of AITC-responsive neurons among KCl-responsive cells was normalized to the vehicle-treated dish from each batch of experiments (**F**). Artemin-exposed neurons showed increased responsiveness to AITC, while the responses to capsaicin and KCl were not impacted (**G-H**). Data are presented as heatmap (**A**), TSNe plot (**B**), means ± SEM (**D, E, H**) or means ± 95% CI of the maximum Fura-2AM (F/F0) fluorescence recorded every 15 seconds (**G**). (**D**) Data from 1 experiment with n=2 technical repeats. (**E**) Pooled data from 2 independent experiments with n=8 technical repeats. (**G**) Representative data from 2 independent experiments with n=107 vehicle-treated and n=122 artemin-treated neurons were analyzed. (**H**) Pooled data from 2 independent experiments with n=4 fields of view per group. Statistical significance is indicated by *p≤0.05, **p≤0.01, ***p≤0.001, ****p≤0.0001.
