# Supplementary material for: NOCICEPTOR NEURONS CONTROL POLLUTION-MEDIATED NEUTROPHILIC ASTHMA
Source: bioRxiv. 2025 Jun 21:2024.08.22.609202. Originally published 2024 Aug 23. Preprint. [Version 2] doi: 10.1101/2024.08.22.609202 (PMC11370576; doi:10.1101/2024.08.22.609202)
Supplement: Supplement 4 [file NIHPP2024.08.22.609202v2-supplement-4.pdf]

bioRxiv preprint doi: https://doi.org/10.1101/2024.08.22.609202; this version posted June 21, 2025. The copyright holder for this preprint (which was not certified by peer review) is the author/funder, who has granted bioRxiv a license to display the preprint in perpetuity. It is made available under aCC-BY 4.0 International license.

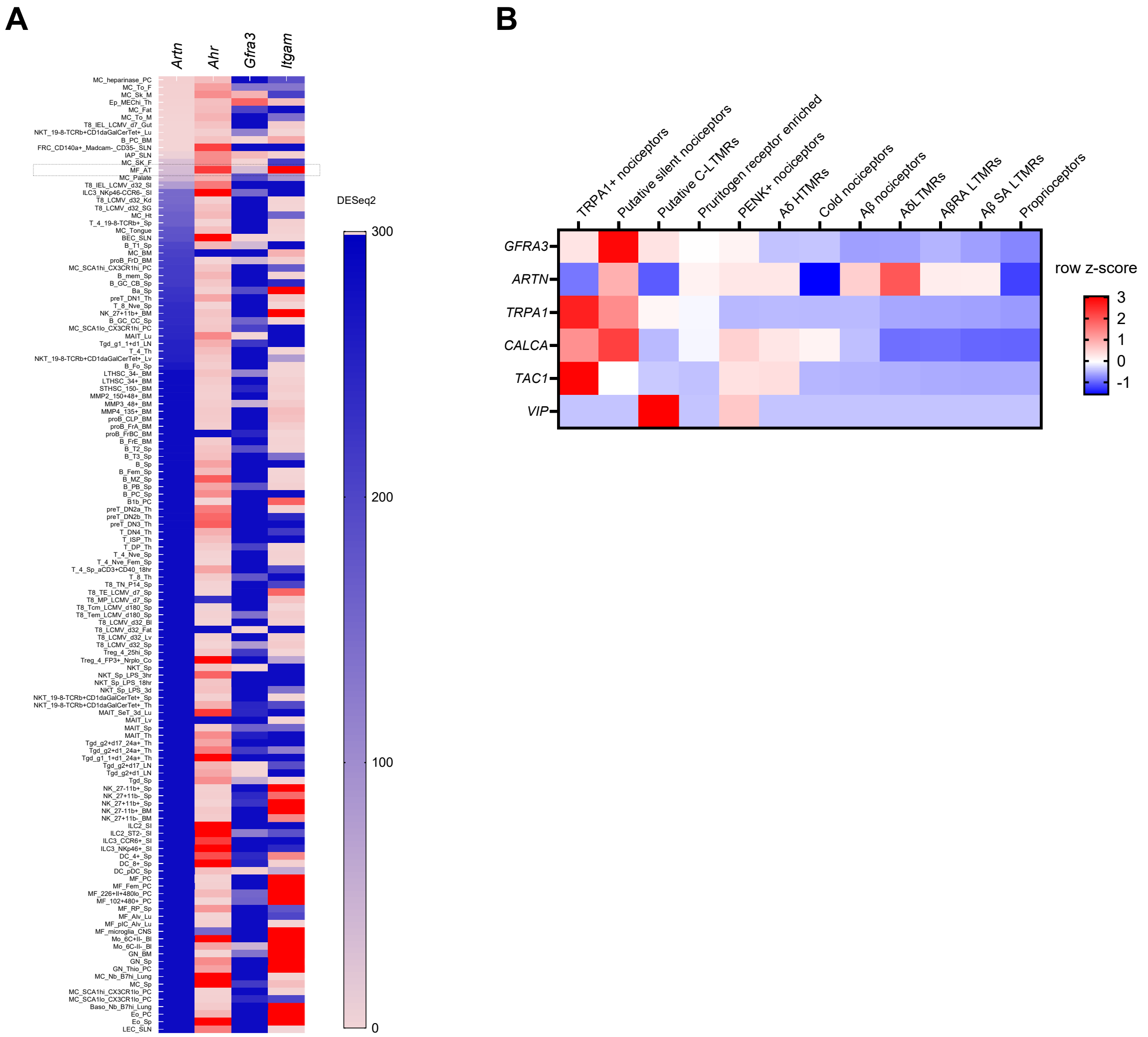

Supplementary Figure 1. In silico analysis of *Artn* expression in mouse immune cells

(**A**) *In-silico* re-analysis of *Artn* expression in mouse immune cells using the ImmGen database[53]. *Artn* and *Ahr* are expressed in *Itgam*⁺ macrophages. Data are presented as per-gene z-scores of normalized gene expression, calculated by the median of ratios method.

(B) *In-silico* re-analysis of the single-cell RNA-seq dataset from Tavares-Ferreira et al., [105] (Sensoryomics; dbGaP accession phs001158) shows that *Gfra3* is expressed in *Trpa1*-positive nociceptors, C-LTMRs, and silent nociceptors within the human dorsal root ganglion. Expression levels are reported as per-gene z-scores calculated with the median-of-ratios normalization method.

bioRxiv preprint doi: https://doi.org/10.1101/2024.08.22.609202; this version posted June 21, 2025. The copyright holder for this preprint (which was not certified by peer review) is the author/funder, who has granted bioRxiv a license to display the preprint in perpetuity. It is made available under aCC-BY 4.0 International license.

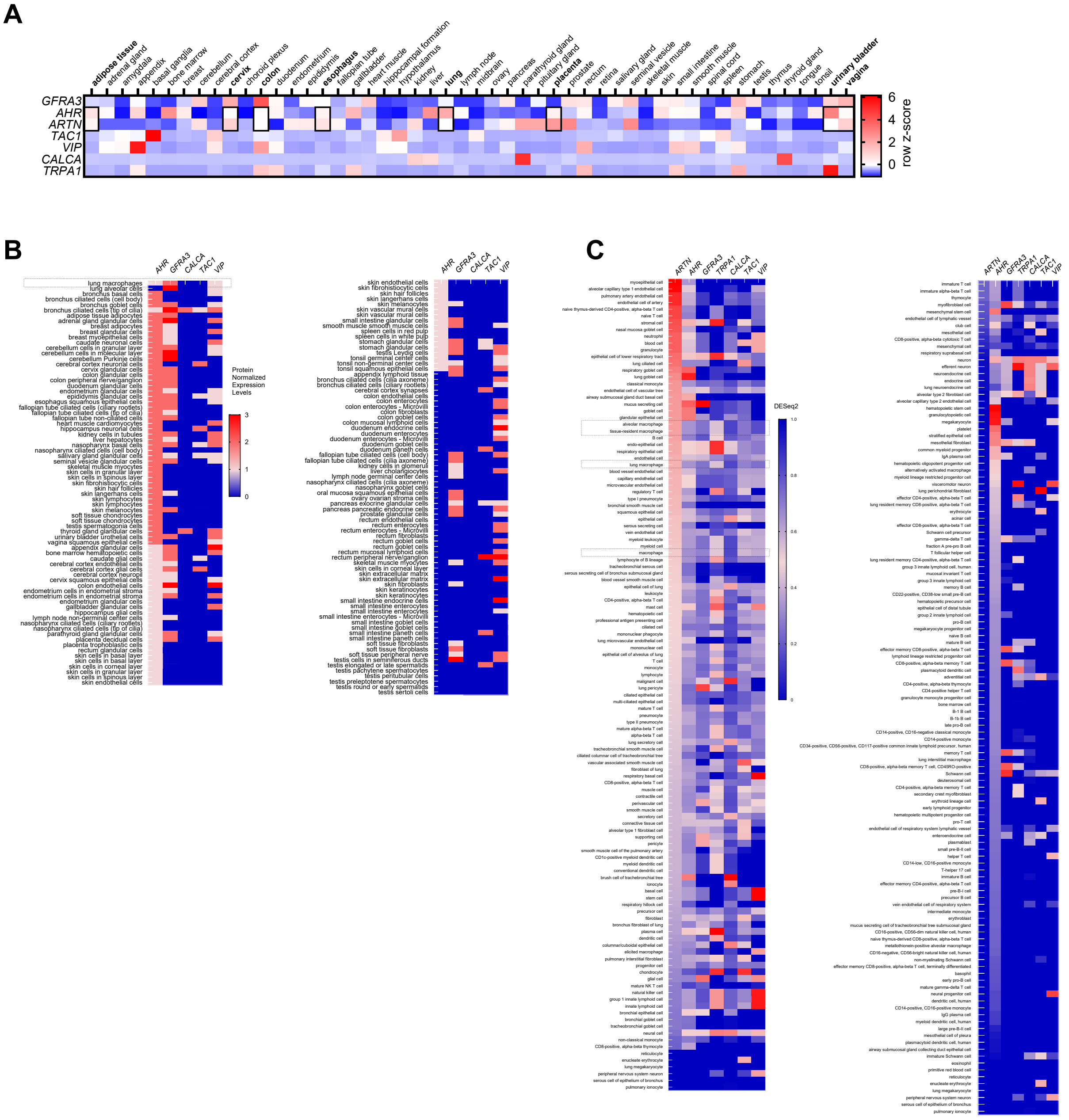

Supplementary Figure 2. *In-silico* re-analysis of Ahr and Artn expression in human tissues

(A) *In-silico* re-analysis of data from Karlsson et al., [106] (Human Protein Atlas, proteinatlas.org) indicates that *Ahr* and *Artn* are expressed in human lung. Expression values are reported as per-gene z-scores using the median-of-ratios normalization method. Experimental details and cell clustering are described by Karlsson et al., [106]

(B) *In-silico* re-analysis of data from Uhlén et al. [55] (Human Protein Atlas, proteinatlas.org) confirms *Ahr* protein expression in human lung macrophages, as shown by immunohistochemistry. These data are presented as protein-normalized expression levels. Experimental details and cell clustering are described by Uhlén et al. [55].

(C) *In-silico* re-analysis of data from Abdulla et al. [56] (CELLxGENE, CZI Single-Cell Biology) reveals co-expression of *Artn* and *Ahr* in lung and alveolar macrophages from patients. Expression values are provided as per-gene z-scores, calculated by the median-of-ratios normalization method. Experimental details and cell clustering are described by Abdulla et al. [56].

bioRxiv preprint doi: https://doi.org/10.1101/2024.08.22.609202; this version posted June 21, 2025. The copyright holder for this preprint (which was not certified by peer review) is the author/funder, who has granted bioRxiv a license to display the preprint in perpetuity. It is made available under aCC-BY 4.0 International license.

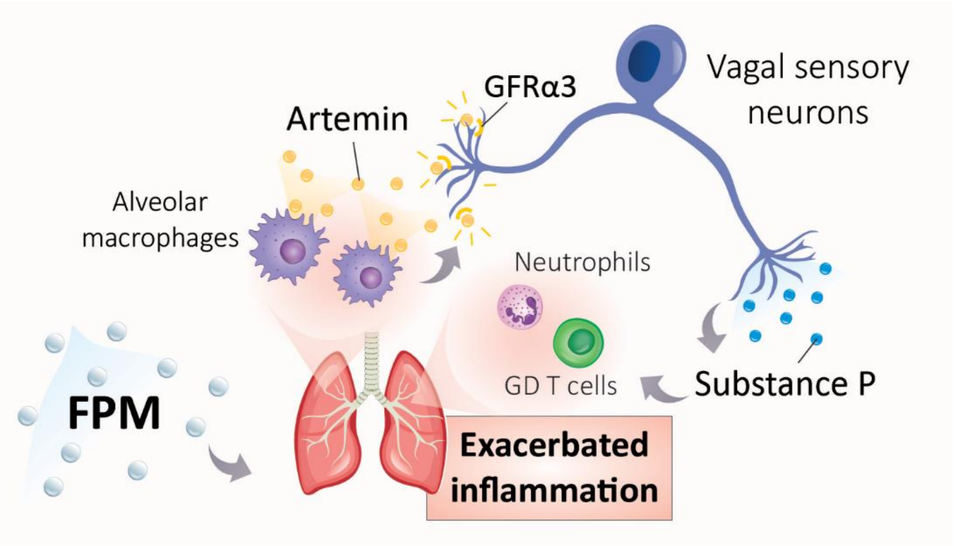

Supplementary Figure 3. Schematic of nociceptor involvement in pollution-exacerbated allergic asthma

In our study, mice were exposed to PM_{25} particles and ovalbumin (OVA) to model pollution-exacerbated asthma. Compared to mice exposed to OVA alone, co-exposure to PM_{25} and OVA significantly increased bronchoalveolar lavage fluid (BALF) neutrophils and lung γδ T cells levels. To counteract this heightened airway inflammation, we administered intranasal QX-314—a charged lidocaine derivative—at the peak of inflammation, effectively normalizing BALF neutrophil levels. Ablation of $TRPV1^+$ nociceptor neurons produced a similar effect.

Further analysis with calcium imaging revealed that neurons from the jugular-nodose complex in pollution-exposed asthmatic mice were more sensitive via their TRPA1 channels. Levels of TNFα and the growth factor artemin were also elevated in the BALF of these mice, returning to normal following nociceptor ablation.

We identified alveolar macrophages as the source of artemin, which they secrete upon sensing fine particulate matter (FPM) through aryl hydrocarbon receptors. Artemin, in turn, heightened TRPA1 responsiveness to its agonist (mustard oil), thereby exacerbating airway inflammation. Our findings suggest that silencing nociceptor neurons can disrupt this pathway, offering a novel therapeutic approach to mitigate neutrophilic airway inflammation driven by pollution.

bioRxiv preprint doi: https://doi.org/10.1101/2024.08.22.609202; this version posted June 21, 2025. The copyright holder for this preprint (which was not certified by peer review) is the author/funder, who has granted bioRxiv a license to display the preprint in perpetuity. It is made available under aCC-BY 4.0 International license.

Supplementary Table 1. Differentially expressed genes and pathway analysis of vagal nociceptors in pollution-exacerbated asthma.

Naïve 6–10 weeks male and female TRPV1^{cre}::tdTomato$^{fl/wt}$ mice were either subjected to a pollution-exacerbated asthma protocol, to the classic ovalbumin (OVA) protocol or remained naïve. On day 17, jugular-nodose complex (JNC) neurons were harvested and dissociated, and TRPV1^{+} (tdTomato^{+}) neurons were FACS-purified to remove stromal and non-peptidergic cells before being processed for RNA sequencing. The different tabs show the DESeq2 identified and analyzed using the web-based tool g:Profiler for each of these conditions. Other tabs show GO terms enriched in each condition.

Supplementary Video 1. Intravital recording of alveolar macrophage motility.

6–10 weeks old male and female littermate control (Na$_V$1.8^{wt}::DTA$^{fl/wt}$ denoted as NaV1.8^{WT}) and nociceptor-ablated (Na$_V$1.8^{cre}::DTA$^{fl/wt}$ denoted as Na$_V$1.8^{DTA}) mice were sensitized via intraperitoneal injection of an emulsion containing ovalbumin (OVA; 200 µg/dose) and aluminum hydroxide (1 mg/dose) on days 0 and 7. On day 10, phagocytes were labeled by intranasal injection of PKH26 (25 pmol/dose). Mice were then challenged intranasally with OVA (50 µg/dose) alone or in combination with fine particulate matter (FPM; 20 µg/dose) on days 14–16. Alveolar macrophage intravital imaging was performed on day 17 and is presented as a 1-hour time-lapse video.

Supplementary Video 2. Intravital recording of neutrophil motility.

Male and female littermate control (Na$_V$1.8^{wt}::DTA$^{fl/wt}$ denoted as Na$_V$1.8^{WT}) and nociceptor-ablated (Na$_V$1.8^{cre}::DTA$^{fl/wt}$ denoted as Na$_V$1.8^{DTA}) mice (6–10 weeks old) were sensitized via an intraperitoneal injection of an ovalbumin (OVA; 200 µg/dose) and aluminum hydroxide (1 mg/dose) emulsion on days 0 and 7. On days 14–16, mice were challenged intranasally with OVA (50 µg/dose) alone or in combination with fine particulate matter (FPM; 20 µg/dose). Immediately before intravital imaging on day 17, an intravenous Ly6G antibody was administered to label neutrophils. The resulting recording is presented as a 20-minute time-lapse video.
