# Supplementary material for: NOCICEPTOR NEURONS CONTROL POLLUTION-MEDIATED NEUTROPHILIC ASTHMA
Source: bioRxiv. 2024 Aug 23:2024.08.22.609202. Preprint. [Version 1] doi: 10.1101/2024.08.22.609202 (PMC11370576; doi:10.1101/2024.08.22.609202)
Supplement: Supplementary file 1 [file NIHPP2024.08.22.609202v1-supplement-1.pdf]

bioRxiv preprint doi: https://doi.org/10.1101/2024.08.22.609202; this version posted August 23, 2024. The copyright holder for this preprint (which was not certified by peer review) is the author/funder, who has granted bioRxiv a license to display the preprint in perpetuity. It is made available under aCC-BY 4.0 International license.

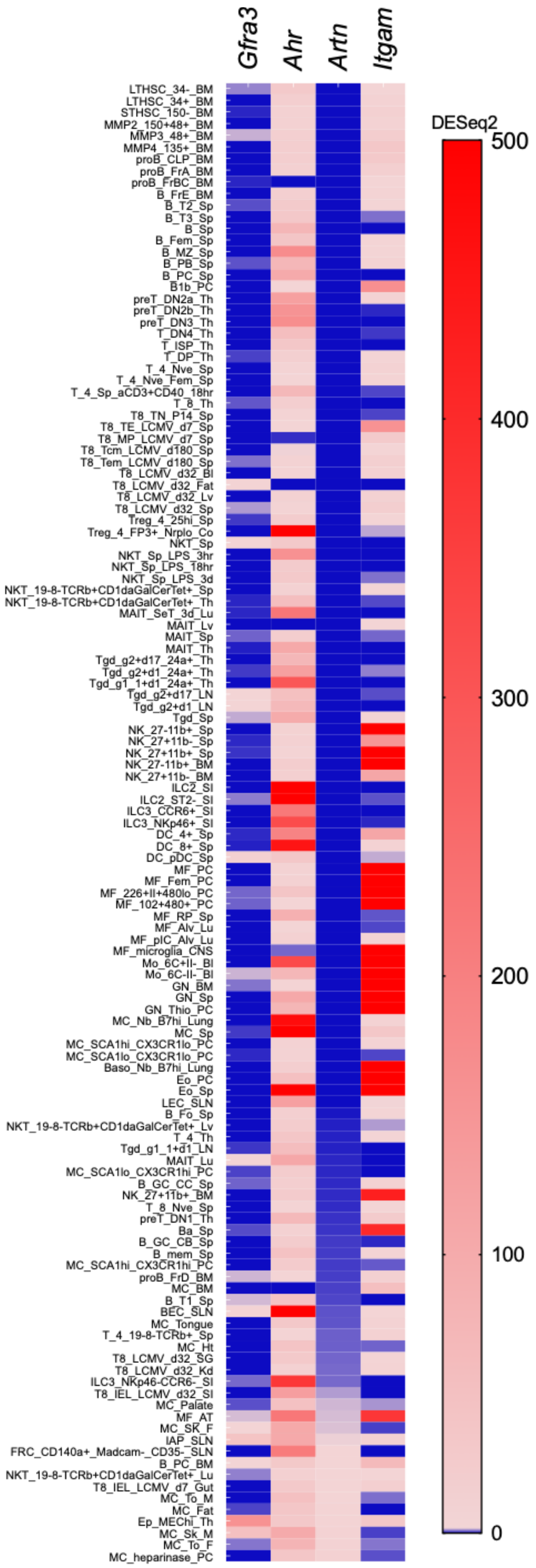

Supplementary figure 1. In silico analysis of Artn expression in mouse immune cells

In silico analysis of Artn expression in mouse using the Immgen database[70].

Data are presented as per-gene z-scores of normalized gene expression using the median of ratios method.

bioRxiv preprint doi: https://doi.org/10.1101/2024.08.22.609202; this version posted August 23, 2024. The copyright holder for this preprint (which was not certified by peer review) is the author/funder, who has granted bioRxiv a license to display the preprint in perpetuity. It is made available under aCC-BY 4.0 International license.

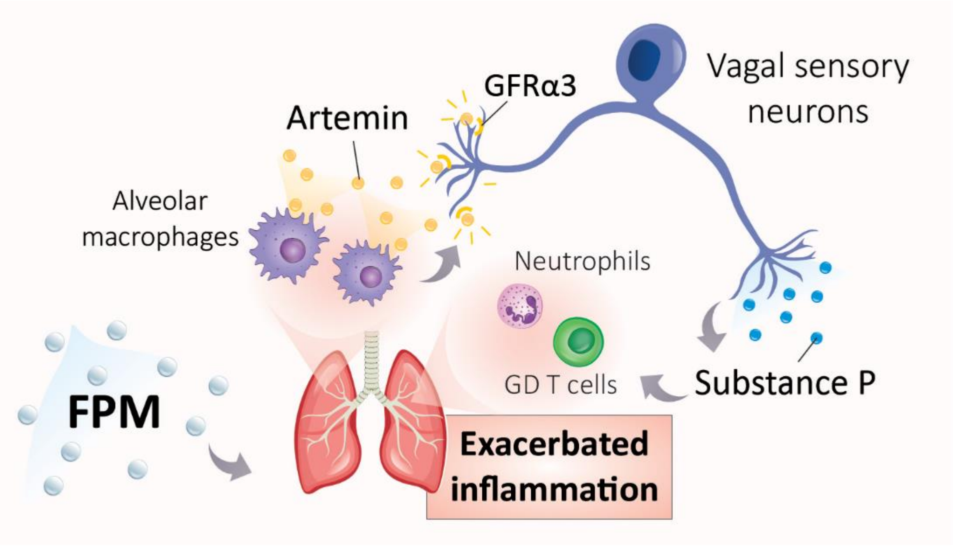

Supplementary figure 2. Schematic of nociceptor involvement in air pollution-exacerbated allergic asthma.

In our study, we modeled pollution-exacerbated asthma in mice by exposing them to $PM_{2.5}$ particles and ovalbumin. This exposure significantly increased bronchoalveolar lavage fluid (BALF) neutrophils and γδ T-cells compared to exposure to ovalbumin alone. To address the heightened airway inflammation, we applied intranasal QX-314, a charged derivative of lidocaine, during peak inflammation, which effectively normalized the inflammation. Similarly, ablating TRPV1-expressing nociceptor neurons also normalized lung neutrophil levels.

Further analysis using calcium microscopy on neurons from the jugular-nodose complex showed increased sensitivity of TRPA1 channels in neurons from pollution-exacerbated asthmatic mice. Elevated TNFα and the growth factor artemin levels were found in the BALF of pollution-exposed mice, which normalized following nociceptor neuron ablation.

We pinpointed alveolar macrophages as the source of artemin, which, expressing aryl hydrocarbon receptors, responded to fine particulate matter (FPM) by releasing artemin. This release increased TRPA1 responsiveness to its agonist, mustard oil, exacerbating airway inflammation. Our findings indicate that silencing nociceptor neurons can interrupt this mechanistic pathway and offer a novel approach to mitigating neutrophilic airway inflammation in pollution-driven asthma.
